# New Possibilities for Hormonal Vaginal Treatment in Menopausal Women

**DOI:** 10.3390/jcm12144740

**Published:** 2023-07-18

**Authors:** Katarzyna Tomczyk, Karolina Chmaj-Wierzchowska, Katarzyna Wszołek, Maciej Wilczak

**Affiliations:** Department of Maternal and Child Health, Poznan University of Medical Sciences, 33 Polna Street, 60-535 Poznań, Poland

**Keywords:** prasterone, estrogen, atrophy

## Abstract

Hormonal vaginal therapy is an effective treatment option for women who experience vaginal symptoms related to hormonal changes. Estrogen and prasterone are widely used as vaginal treatments, particularly for urogenital atrophy. These symptoms may include vaginal dryness, itching, burning, and pain during sexual intercourse, all of which can significantly affect a woman’s quality of life. Previous studies have indicated that such treatment improves tissue elasticity, moisturizes the vagina, and can have a substantial impact on urine incontinence and vaginal microflora and decreases dyspareunia. Hormonal therapy is also useful and commonly used before vaginal surgical treatment. Prasterone is quite a new option for vaginal therapy in Poland and is mainly recommended for dyspareunia in menopausal women. The study related to prasterone therapy emphasizes its effectiveness and safety, making it advantageous to explore its beneficial impact. This paperwork aims to summarize the mechanism of action as well as the effects of both drugs and their beneficial action during vaginal treatment.

## 1. Introduction

Hormonal vaginal therapy is an effective treatment option for women who experience vaginal symptoms related to hormonal changes. These changes are mainly observed in the menopause period, which commonly occurs after natural ovarian failure. Menopause typically occurs around the age of 50, but the timing can vary for each woman. It marks the transition from reproductive to nonreproductive years and is characterized by the absence of menstrual periods. Hormonal changes can also be observed in women with premature ovarian failure, which occurs before the age of 40, as well as in cases of ovarian failure resulting from oncologic therapies like chemotherapy and radiotherapy.

These hormonal fluctuations can lead to unpleasant vaginal symptoms, such as dryness, itching, burning, and pain during sexual intercourse, significantly impacting a woman’s quality of life. Vulvovaginal atrophy (VVA) is a condition that arises after menopause when estrogen levels are extremely low due to ovarian failure. Women may experience various distressing symptoms during this time, including dryness of the vagina, dyspareunia, itching, irritation, urine incontinence, and urinary tract infections (UTIs) [1]. All of those symptoms are indications for vaginal hormonal treatment.

In 2014, the International Society for the Study of Women’s Sexual Health and the NAMS introduced a new term for VVA—genitourinary syndrome of menopause (GSM) [2]. The aim was to broaden the definition to cover the scale of the problem involving changes to the whole urogenital tissues responsive to both estrogens and androgens [2]. GSM is a prevalent disorder, potentially affecting up to three-quarters of menopausal women [3].

Estrogen and prasterone, androgenic options, are widely used as a vaginal treatment in those cases. Those medicaments are applied, especially for urogenital atrophy [4].

In the treatment of the menopausal period, we can distinguish between systemic and local vaginal therapy [5]. Often, both types of therapy are necessary, especially when patients experience common menopausal symptoms, such as hot flashes, chills, night sweats, sleep problems, mood changes, weight gain, and a slowed metabolism. Systemic hormonal therapy involves the administration of estrogens, and for women with a uterus, progesterone is additionally given to prevent endometrial hyperplasia. Systemic oral therapy contains higher doses of estrogens than vaginal therapy, and substituting the missing hormones affects those systemic symptoms. The mechanism of action is the same for both applying ways of these drugs. However, the systemic application enables systemic action, while a local supply affects mainly local symptoms, as local vaginal therapy is directly applied to the vaginal area and is recommended for women who experience only vaginal symptoms. This therapy can help restore normal vaginal thickness, mucosa moisture, and elasticity, thereby alleviating vaginal symptoms [6].

Local vaginal therapy comprises moisturizing gels and hormonal substances, such as estrogen vaginal tablets and gels. A new alternative in this category is vaginal tablets containing prasterone [7]. Hormonal vaginal therapy proves beneficial for VVA and is commonly used before planned vaginal surgeries. It is believed to facilitate the preparation of vaginal tissues and increase surgical safety. Additionally, it may be advantageous in cases involving mesh implantation, as it can reduce the risk of cystitis and bacteriuria following surgery [8,9]. However, there is currently no clear data regarding the real impact of local hormonal therapy on reducing tearing and bleeding during gynecological surgeries, avoiding blood transfusions, improving tissue healing, and facilitating postoperative recovery [10]. Similarly, there is no definitive information regarding the perfect duration of treatment that would yield the most benefits [10,11]. It appears that each gynecological center has its hormonal recommendations for preparing vaginal tissues before vaginal surgeries, especially those involving mesh implantation.

The goal of this paperwork is to provide an overview of the mechanisms of action of both available estrogen and prasterone when administered vaginally. The paperwork refers mainly to short-term therapy, as it seeks to conclude the effects and benefits of such treatment.

## 2. Methods

We searched the Web of Science Core Collection, National Library of Medicine (PubMed), and the Cochrane Database, using the following phrases “VAA” or “GSM”, “Estrogenes” or “Prasterone”, “vaginal microbiota”, “vaginal mesh”, “stress urinary incontinence”, “VMI”, or “menopause”. The search spanned from 1980 to the current date. We carefully reviewed the titles and abstracts of the retrieved articles and selected those that were relevant to the topic at hand.

## 3. Estrogens

According to Cochrane analyses, estrogenic products are significantly more beneficial than nonhormonal gels as a treatment for VAA [12]. It is well acknowledged that vaginal estrogens have various positive effects, such as improving the vaginal maturation index (VMI), reducing vaginal pH, enhancing vaginal muscle tone, promoting subepithelial capillary growth, and increasing the number of vaginal sensory nerves [13,14]. Two types of vaginal estrogen treatments are commonly used: estradiol and estriol, which are available in the form of tablets and creams. However, there is currently no comprehensive data or recommendations regarding the use of estrogens before pelvic organ prolapse (POP) surgeries [15]. Nevertheless, an increase in vaginal epithelial thickness has been observed, which could potentially enhance the effectiveness of mesh implants [8,16]. Due to this observation, many clinicians adjust the dosage and time of vaginal estrogen therapy on an individual basis.

Published data have provided insights into the observed changes in the vagina following the preoperative use of estradiol. Fedding et al. conducted a study where 25 mcg of estradiol was administered vaginally for 3 weeks before POP surgery. They reported improved VMI and epithelial thickness, as well as a lower frequency of UTIs compared to a placebo tablet used before surgery [8]. In the study by Vaccaro et al., patients used 1 g of nightly conjugated estrogens (0.625 mg/g) for 2–12 weeks before surgery. They observed better VMI with the treatment but no significant changes in epithelial thickness [1]. Another noteworthy study concluded that after 6–8 weeks of using the 0.625 mg/g conjugated estrogens cream (1 g nightly), there was an increase in epithelial thickness while the subepithelial thickness remained unchanged [16]. The published literature indicates that VMI increases by approximately 15% when vaginal estrogens are used for 2–12 weeks before surgery [1].

The VMI is an index that describes the proportion of three types of vaginal cells: parabasal, intermediate, and superficial cells. This proportion is influenced by the hormonal status of the patients. The total score of VMI ranges from 0 to 100%, with different ranges indicating varying levels of estrogenic effect. A VMI score of 0–49% suggests an absent or low estrogenic effect, 50–64% indicates a moderate estrogenic effect, and 65–100% is typical for a high estrogenic effect [17]. It has been suggested that a low VMI score is associated with the occurrence and severity of GSM in menopausal women [18]. Other interesting results referring to short estrogenic vaginal treatment are presented in Table 1.

Estrogens affect the estrogen receptors (ER) found throughout the lower urinary tract. However, during menopause, this mechanism is affected by a decrease in ER-b levels and an increase in ER-a levels. This alteration may contribute to stress incontinence [29,30,31]. Estrogens exhibit the following mechanisms of action: enhancement of the cellular lining of the vagina, urethra, and bladder by increasing cell count; improvement in periurethral vascularity and urethral pressure [32,33,34]. Furthermore, they impact the genitourinary system by modifying the quantity and quality of the mucopolysaccharide layer that lines the bladder and urethra, increasing the visceral smooth muscle tone and sympathetic nerve density in the pelvis and regulating neurotrophins [35]. All of these mechanisms appear to be responsible for maintaining a stable pelvic floor and promoting proper urination, thus consequently reducing the risk of both stress urinary incontinence (SUI) and an overactive bladder.

These mechanisms could potentially be beneficial in the context of POP surgeries. It is worth noting that the vaginal dose of estrogens is typically ten times lower than the oral dose, resulting in plasma levels that remain within the normal postmenopausal range of 50 pmol/L [36,37]. This localized treatment approach helps to minimize the systemic side effects. However, there is still a lack of published studies confirming the extent of these benefits [38]. It is widely believed that despite the local application, the drug can still lead to systemic side effects, potentially contributing to cardiovascular and cerebrovascular diseases, estrogenic cancers, and thromboembolic complications [5].

## 4. Prasterone

In Poland, a recent vaginal treatment option for VAA in menopausal women has emerged, involving the use of an androgen called dehydroepiandrosterone (DHEA). Androgens are produced in equal amounts by the adrenal glands and ovaries in women of childbearing age. Testosterone is predominantly an ovarian hormone, while DHEA is primarily of adrenal origin [39]. However, starting at around the age of 30, androgen levels in women begin to decline, and after menopause, they are observed at only 10–20% of their previous levels [4,40]. DHEA appears to function through multiple signaling pathways as a neurosteroid and indirectly affects sex-steroid target tissues [41,42].

**Figure 1:**
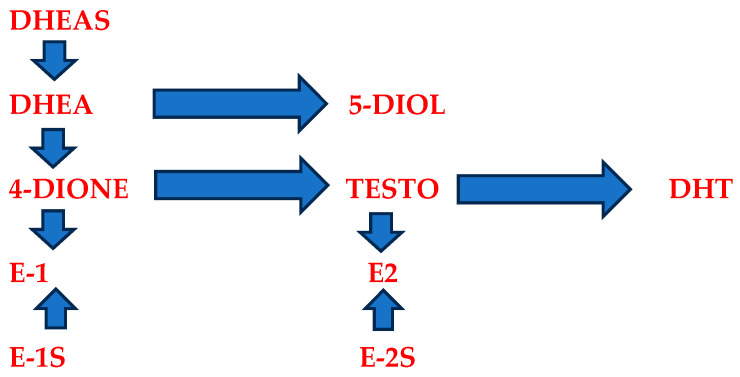
The enzymatic conversion of prasterone. DHEAS—dehydroepiandrosterone sulfate; DHEA—dehydroepiandrosterone; DHT—dehydrotestosterone; 4-DIONE—androstenedione; E1—estrone; E1S—estrone sulfate; 5-DIOL—androst-5-ene-3β; TESTO—testosterone; E2—17-betaestradiol; E-2S—17-betaestradiol sulfate.

As exogenous DHEA is metabolized similarly to endogenous DHEA, it is converted into testosterone and estrogens, thereby exerting an indirect estrogenic effect with potentially fewer systemic effects [43,44]. The enzymatic conversion of prasterone is schematically shown in Figure 1.

Studies indicate that intravaginal treatment with DHEA does not lead to an increase in blood levels of sex steroids, such as estradiol, DHEA, DHEAS, androstenedione, and testosterone [45]. The advantageous aspect of intravaginal DHEA is its ability to act through both estrogenic and androgenic actions [46]. One of its main effects on tissues is the stimulation of collagen production and increased density in the lamina propria [47,48]. Importantly, it has shown effectiveness in all three layers of the vagina: the epithelium, lamina propria, and muscularis [49]. By improving VMI, vaginal elasticity, and lubrication, this treatment benefits menopausal women. In 2017, the FDA approved vaginal DHEA (prasterone (Intrarosa)) for the treatment of dyspareunia caused by VVA. This remains the primary indication for such treatments, as they do not affect sexual desire or other sexual dysfunctions in women [4].

In the case of vaginal treatment, it appears that the levels of aromatase (the enzyme that converts androgen to estrogen) expressed in the endometrium are minimal, indicating a low potential risk of endometrial hyperplasia or cancer. Short-term supplementation studies have shown no significant effect on the endometrium [50,51]. However, there is limited data on the long-term safety of vaginal DHEA, particularly regarding cardiovascular diseases and the risk of breast cancer. Prasterone has not been studied in breast cancer survivors, and its use is contraindicated in individuals with current or past breast cancer [52]. Due to the lack of clinical efficacy and long-term safety data, the Endocrine Society advises against extensive long-term use of DHEA in women [53].

Contraindications for using this hormone include acute liver disease, undiagnosed genital bleeding, breast cancer, estrogen-dependent cancers, untreated endometrial hyperplasia, venous thromboembolism, thrombophilic disorders, arterial thromboembolic disease, and porphyria [54].

The published results of using prasterone in GSM are presented in Table 2.

## 5. Vaginal Microbiota and Hormonal Vaginal Therapy

Regarding hormonal vaginal therapy, it is required that we mention its impact on the vaginal microflora. In the case of GSM symptoms, supporting the correct microflora seems to be of great importance and helps to combat the mentioned unpleasant postmenopausal symptoms.

The vaginal microbiota is a complex ecosystem that significantly influences the overall health of the female reproductive system [56]. It consists of a diverse range of microorganisms, with bacteria being the most prominent constituents [57,58]. Among the bacteria, *Lactobacillus* species, including *Lactobacillus crispatus*, *Lactobacillus iners*, *Lactobacillus jensenii*, and *Lactobacillus gasseri*, are the predominant bacteria found in a healthy vaginal microbiota [56]. These beneficial bacteria play a vital role in maintaining vaginal health through the production of lactic acid, hydrogen peroxide, and bacteriocins [59], which help maintain an acidic pH. The acidic environment created by *Lactobacillus* species inhibits the growth of harmful pathogens. Additionally, a healthy vaginal microbiota adheres to the vaginal epithelium, preventing the adhesion of other bacteria [60], and regulates immune and inflammatory responses, thereby enhancing the vaginal resistance to diseases [61]. However, during menopause, decreased levels of estrogen secretion lead to vaginal atrophy and reduced glycogen levels, resulting in a decline in vaginal lactobacilli counts [62].

Vaginal dysbiosis, characterized by changes in the vaginal pH, is often associated with an increased risk of various vaginal infections, including bacterial vaginosis, vulvovaginal candidiasis, human papillomavirus infection, genital herpes infection, and other sexually transmitted infections [63,64].

Hormonal vaginal therapy is a treatment approach that utilizes hormone-based treatments to address specific concerns related to vaginal health, particularly those associated with menopause [6,65]. By restoring the estrogen levels in vaginal tissues, hormonal vaginal therapy helps improve vaginal lubrication, elasticity, and overall vaginal health [6]. It can reduce discomfort during intercourse, alleviate vaginal dryness, and reduce the risk of UTIs.

The vaginal microbiota and hormonal vaginal therapy have interrelated implications for women’s health. Firstly, changes in the vaginal microbiota can impact the effectiveness of hormonal vaginal therapy. A healthy vaginal microbiota with an optimal balance of *Lactobacillus* species provides a favorable environment for hormonal treatments to work efficiently. Estrogen, when used intravaginally, reduces unpleasant vaginal symptoms and allows it to stimulate the renewal and secondary colonization of the vagina with Lactobacillus bacteria. It also contributes to the deposition of glycogen in the vaginal epithelium, which is metabolized by local bacterial communities to produce the organic acids needed to protect the genital tract. Postmenopausal women undergoing hormonal treatment have been found to have significantly higher levels of free glycogen and *Lactobacillus* spp. compared to those not using hormonal treatment [66]. However, the presence of a Lactobacillus-dominant vaginal microbiota is not necessarily associated with fewer vulvovaginal symptoms [67]. While serum estrone levels are higher in women with Lactobacillus dominance, the composition of the vaginal microbiota is not associated with vaginal free glycogen levels [67].

On the other hand, imbalances in the vaginal microbiota, such as in the case of BV, can affect the absorption and efficacy of hormone-based therapies [56,68].

Conversely, hormonal changes can also have an impact on the composition and health of the vaginal microbiota. Estrogen, for example, plays a role in promoting the growth of *Lactobacillus* species and helps maintain a healthy vaginal environment [69]. As estrogen levels decline during menopause, there is a higher risk of imbalances in the vaginal microbiota and related symptoms.

Furthermore, emerging research suggests that there is an interconnected relationship between the vaginal and gut microbiota, which can have implications for overall health [56,59]. Alterations in the gut microbiota can impact vaginal health, potentially affecting the risk of infections or imbalances in the vaginal microbiota [56,59]. Similarly, disruptions in the vaginal microbiota can influence the gut microbiota, although the mechanisms and implications of this interplay are still being explored.

## 6. Discussion

Vaginal estrogen therapy is beneficial in treating symptoms of VVA. There is a lot of evidence supporting its effectiveness in addressing symptoms such as dyspareunia, dryness, itching, and cystitis [70]. The molecular mechanism underlying its action has been well described and confirmed in numerous studies.

However, the efficacy of preoperative vaginal estrogen treatment in postmenopausal women undergoing POP surgery remains uncertain. A Cochrane review found no clear data indicating that estrogens can help reduce symptoms of POP [12]. A study by M-L Marschalek et al. revealed that 6 weeks of vaginal estrogen therapy before POP surgery yielded the same results as a placebo treatment. POP symptoms, bowel and bladder functions, as well as sexual function were assessed similarly in both groups [71]. Additionally, Zhixing Sun et al. conducted a one-year follow-up after mesh implantation for POP in postmenopausal women. Their findings showed no significant differences in mesh exposure between women treated with vaginal estrogens and those given a placebo [72].

Xia Yu et al., in their systematic review, summarized the findings from seven randomized controlled trials investigating the impact of local estrogen treatment in women with POP compared to women treated with a placebo. Their review concluded that there was no significant improvement in epithelial thickness, vaginal pH, and quality of life questionnaire scores. However, a slight increase in VMI was observed [73].

Regarding SUI, estrogen vaginal treatment is recommended as one method of conservative therapy. If conservative measures fail, surgical treatment may be suggested [74]. However, the literature does not provide data on the optimal timing of using local vaginal treatment before sling surgeries or whether such management is beneficial in the case of potential future sling erosion.

Similarly, there is a lack of data regarding the effects of short-term vaginal prasterone treatment before surgeries for urinary incontinence or POP. However, in the treatment of urge incontinence, Claudia Collà Ruvolo et al. presented interesting findings. Their study suggested that 12 weeks of prasterone treatment may have a positive impact on the severity of urinary urge incontinence in women, although it should be noted that the study was conducted on a small group of patients. It is worth noting that 89.7% of women reported a reduction in the discomfort of urgency in the prasterone group compared to the hyaluronic acid group, as assessed by the Patient Global Impression of Improvement score ≤3 [75]. However, there is currently no available data on the effect of short-term local vaginal prasterone treatment for stress urinary incontinence.

In the literature, there are available data on the use of preoperative estrogen therapy, but currently, no data on the use of prasterone before POP surgery. Therefore, we do not have information regarding the benefits experienced by patients, or the tissue changes observed with short-term prasterone therapy in this context.

Vaginal therapy with prasterone is primarily recommended for improving sexual disorders in menopausal women. In a study by Labrie et al., women received 6.5 mg of prasterone vaginally for 12 weeks, and the authors found that all components of the Female Sexual Function Index showed significant improvement. These components included desire, arousal, lubrication, orgasm, sexual satisfaction, and pain during sexual activity [49]. Consequently, prasterone is commonly prescribed as a treatment for dyspareunia.

Indeed, it would be advantageous to understand the effects of local vaginal treatment with prasterone, especially before gynecological vaginal procedures. Estrogenic vaginal treatments have been known to affect endometrial thickness and can potentially lead to endometrial hyperplasia. In a study by Felding et al., the use of preoperative estradiol tablets (25 µg) for only 3 weeks resulted in simple endometrial hyperplasia on the day of the surgery [8]. Similarly, in the Riou study, the application of conjugated equine estrogen cream at a dose of 2 g daily for 21 days, followed by a seven-day break, and then a repeated regimen for 6 months resulted in endometrial thickness disorders. Two patients showed endometrial hyperplasia, seven patients had proliferative endometrium, and four patients had a histopathological diagnosis of weakly proliferative endometrium [37].

Considering these findings, it would be beneficial to investigate whether short-term local vaginal therapy with prasterone has similar adverse effects.

Another interesting issue to consider is examining the impact of short-term local vaginal therapy using prasterone on vaginal bacterial flora. The combination of hormonal estrogenic therapy and lactobacilli appears to have beneficial effects on GSM. It is claimed that the lack of estrogen has a negative effect on vaginal microbiota. Therefore, it is necessary to supplement both types of therapy to prevent infectious and menopausal symptoms, such as itching, swelling, and dyspareunia [66]. Postmenopausal women are observed to have a lower diversity in vaginal bacterial flora, mainly in the Lactobacilli type, as well as a lower amount of these protective bacteria. It is also suggested that women on hormonal replacement therapy have much better microflora compared to those without such treatment [66]. On the other hand, an increasing number of studies demonstrate that postmenopausal dysbiosis, rather than a low estrogenic effect, might be the cause of GSM symptoms, such as vaginal dryness, itching, and recurrent urinary infections [66,76]. In a study by Hummelen, women with severe VVA symptoms showed a decreased amount of *Lactobacillus* species and greater bacterial vaginal diversity. Among them, *Prevotella*, *Porphyromonas*, *Peptoniphilus*, and *Bacillus* were distinguished [76]. Interestingly, there is no available data on combined vaginal therapies involving prasterone. Furthermore, there are currently no medications that combine both prasterone and *Lactobacillus*.

The vaginal microbiota and hormonal vaginal therapy play crucial roles in women’s health. A well-balanced vaginal microbiota is vital for preserving vaginal health and preventing infections. By comprehending the intricate interactions between the vaginal microbiota and hormonal therapy, the treatment approaches can be improved, and outcomes thus optimized. Furthermore, investigating the interconnectedness between the vaginal and gut microbiota holds great potential for advancing research in women’s health and developing personalized healthcare strategies.

## 7. Conclusions

Local vaginal therapy is advantageous for women experiencing GSM symptoms. The positive effects of vaginal estrogens, as well as the combination of estrogens with lactobacilli species, are well-established. There is substantial evidence confirming that this type of therapy improves various menopausal vaginal disorders, including vaginal dryness, itching, dyspareunia, vaginal elasticity, and lubrication. However, there is still limited data regarding the use of prasterone in such therapy. Prasterone is an androgen that can act directly and indirectly by converting into estrogens. Currently, prasterone is mainly recommended for the treatment of dyspareunia; however, it appears that this medication may have additional beneficial impacts with a high level of safety.

## 8. Future Directions

This study serves as an announcement for evaluating the effect of prasterone on vaginal tissues, systemic hormonal levels, endometrial thickness, and vaginal microbiota in patients with urogynecological problems, particularly those experiencing GSM symptoms. The data collected from this study may also aid in better preparing patients for urogynecological surgeries, considering both the safety and benefits of preoperative vaginal treatment. It is essential to determine whether this type of vaginal therapy can be beneficial in terms of mesh implantation and preventing potential erosion.

This study has its limitations. There is little data about the medical effects of prasterone, and the obtained data are mainly from women with dyspareunia, unlike the estrogen therapy widely used in women with GSM symptoms as well as with pelvic organ prolapse.

## Figures and Tables

**Table 1:** Impact of estrogens on the genitourinary syndrome of menopause—a literature review.

Paperwork	Dose	Time of Treatment	*n*	VMI	Vaginal pH	Lubrication	Dyspareunia	UTI
Bauchmann 2008 [19]	0.3 mg estrogen (21 days on/7 days off or twice weekly)	12 weeks	423	+	+	+	?	?
Cano 2012 [20]	50 mcg estriol Daily for 3 weeks and then twice weekly	12 weeks	114	+	+	+	?	?
Dessole 2004 [21]	Intravaginal oestradiol ovules (1 mg) once daily for 2 weeks and then two ovules once weekly for 6 months	6 months	44	?	+	+	+	+
Griesser 2012 [22]	Estriol pessary 0.2 mg. Once daily for 20 days and twice weekly for 9 weeks.	12 weeks	142	+	+	+	?	?
	Estriol pessary 0.03 mg. Once daily for 20 days and twice weekly for 9 weeks.		147	+	+	+	?	?
Karp 2012 [23]	Oestradiol-releasing vaginal ring	12 weeks	22	+	+	+	?	?
Raghunandan 2010 [24]	0.625 mg of conjugated equine estrogen for 2 weeks and twice weekly for 10 weeks	12 weeks	25	+	+	+	+	?
Daneshmand 2014 [25]	Vaginal estrogen cream 1 tube per night for 14 nights, then 1 tube 2 nights in 1 week for 10 weeks	12 weeks	80	?	?	+	+	
	Estrogen tablet 25 mcg tablets	12 weeks	80	?	?	+	+	?
Dugal 2000 [26]	Oestradiol vaginal tablet 25 mcg 17β oestradiol	12 weeks	85	?	?	+	+	?
Barentses 1997 [27]	Oestradiol ring containing 2 mg micronized 17β oestradiol with a constant release of 7.5 mcg oestradiol/24 h or estriol cream containing 1 mg estriol/G of cream 0.5 mg daily for 2 weeks followed by maintenance dose 0.5 mg	12 weeks	165	+	+	+	+	?
Casper 1999 [28]	Oestradiol-releasing silicone ring	24 weeks	33	+	+	+	+	?

+, improvement; ?, no data; *n*, number of patients; VMI, vaginal maturation index; UTI, urinary tract infection.

**Table 2:** Impact of prasterone on the genitourinary syndrome of menopause—a literature review.

Paperwork	Dose	Time of Treatment	*n*	VMI	Vaginal Ph	Lubrication	Sexual Function	UTI
Labrie 2009 [55]	Intravaginal prasterone (0.25, 0.5, 1.0%)	12 weeks	216	+	+	+	+	?
Archer [13]	Intravaginal prasterone 0.50%	12 weeks	253	+	+	+	+	?
Labrie [49]	Intravaginal prasterone 0.50%	12 weeks	325	+	+	+	+	?
Labrie [46]	Intravaginal prasterone 0.50%	Up to 52 weeks	530	+	+	?	?	?

+, improvement; ?, no information; *n*, number of patients; VMI, vaginal maturation index; UTI, urinary tract infection.

## Data Availability

Data are unavailable on request due to restrictions, e.g., privacy or ethics.

## References

[1] Vaccaro C.M., Mutema G.K., Fellner A.N., Crisp C.C., Estanol M.V., Kleeman S.D., Pauls R.N. (2013). Histologic and cytologic effects of vaginal estrogen in women with pelvic organ prolapse: A randomized controlled trial. Female Pelvic Med. Reconstr. Surg..

[2] Portman D.J., Gass M.L. (2014). Vulvovaginal Atrophy Terminology Consensus Conference Panel. Genitourinary syndrome of menopause: New terminology for vulvovaginal atrophy from the International Society for the Study of Women’s Sexual Health and the North American Menopause Society. Menopause.

[3] Palma F., Volpe A., Villa P., Cagnacci A. (2016). Vaginal atrophy of women in postmenopause. Results from a multicentric observational study: The AGATA study. Maturitas.

[4] Vegunta S., Kling J.M., Kapoor E. (2020). Androgen Therapy in Women. J. Womens Health (Larchmt).

[5] Kagan R., Kellogg-Spadt S., Parish S.J. (2019). Practical Treatment Considerations in the Management of Genitourinary Syndrome of Menopause. Drugs Aging.

[6] Krause M., Wheeler T.L., Snyder T.E., Richter H.E. (2009). Local Effects of Vaginally Administered Estrogen Therapy: A Review. J. Pelvic Med. Surg..

[7] Rutkowski K., Sowa P., Talipska-Rutkowska J., Kuryliszyn-Moskal A., Rutkowski R. (2014). Dehydroepiandrosterone (DHEA): Hypes and hopes. Drugs.

[8] Felding C., Mikkelsen A.L., Clausen H.V., Loft A., Larsen L.G. (1992). Preoperative treatment with oestradiol in women scheduled for vaginal operation for genital prolapse. A randomised, double-blind trial. Maturitas.

[9] Mikkelsen A.L., Felding C., Clausen H.V. (1995). Clinical effects of preoperative oestradiol treatment before vaginal repair operation. A double-blind, randomized trial. Gynecol. Obs. Obstet. Investig..

[10] Maher C., Feiner B., Baessler K., Glazener C.M.A. (2010). Surgical management of pelvic organ prolapse in women. Cochrane Database Syst. Rev..

[11] Rees M., Pérez-López F.R., Ceasuc I., Depyperee H., Erelf T., Lambrinoudaki I., Schenck-Gustafssonh K., Simoncinii T., van der Schouwj Y.T., Tremollieres F. (2012). EMAS clinical guide: Low-dose vaginal estrogens for postmenopausal vaginal atrophy. Maturitas.

[12] Lethaby A., Ayeleke R.O., Roberts H. (2016). Local oestrogen for vaginal atrophy in postmenopausal women (Review). Cochrane Libr..

[13] Archer D.F., Labrie F., Bouchard C., Portman D.J., Koltun W., Cusan L., Labrie C., Côté I., Lavoie L., Martel C. (2015). Treatment of pain at sexual activity (dyspareunia) with intravaginal dehydroepiandrosterone (prasterone). Menopause.

[14] Griebling T.L., Liao Z., Smith P.G. (2012). Systemic and topical hormone therapies reduce vaginal innervation density in postmenopausal women. Menopause.

[15] Rahn D.D., Ward R.M. (2015). Vaginal estrogen use in postmenopausal women with pelvic floor disorders: Systematic review and practice guidelines. Int. Urogynecol. J..

[16] Rahn D.D., Good M.M., Roshanravan S.M., Shi H., Schaffer J.I., Singh R.J., Word R.A. (2014). Effects of preoperative local estrogen in postmenopausal women with prolapse: A randomized trial. J. Clin. Endocrinol. Metab..

[17] Marx P., Schade G., Wilbourn S., Blank S., Moyer D.L., Nett R. (2004). Low-dose (0.3 mg) synthetic conjugated estrogens a is effective for managing atrophic vaginitis. Maturitas.

[18] Lumbanraja I.L., FGSiregar M.F.G., Lumbanraja S.N., Adenin I., Lintang L.S., Halim B. (2021). Association of Vaginal Maturation Index and Vaginal pH with the Most Bothersome Symptoms of Genitourinary Syndrome of Menopause. J. South Asian Fed. Obstet. Gynaecol..

[19] Bahmann G., Bouchard C., Hoppe D., Ranganath R., Altomare C., Vieweg A., Graepel J., Helzner E. (2009). Efficacy and safety of low-dose regimens of conjugated estrogens cream administered vaginally. Menopause J. N. Am. Menopause Soc..

[20] Cano A., Estévez J., Usandizaga R., Gallo J.L., Guinot M., Delgado J.L., Castellanos E., Moral E., Nieto C., del Prado J.M. (2012). The therapeutic effect of a new ultra low concentration estriol gel formulation (0.005% estriol vaginal gel) on symptoms and signs of postmenopausal vaginal atrophy: Results from a pivotal phase III study. Menopause.

[21] Dessole S., Rubattu G., Ambrosini G., Gallo O., Capobianco G., Cherchi P., Marci R., Cosmi E. (2004). Efficacy of low-dose intravaginal estradiol on urogenital aging in postmenopausal women. Menopause.

[22] Griesser H., Skonietzki S., Fischer T., Fielder K., Suesskind M. (2012). Low dose estriol pessaries for the treatment of vaginal atrophy: A double-blind placebo-controlled trial investigating the efficacy of pessaries containing 0.2 mg and 0.03 mg estriol. Maturitas.

[23] Karp D.R., Jean-Michel M., Johnston Y., Suciu G., Aguilar V.C., Davila G.W. (2012). A randomized clinical trial of the impact of local estrogen on postoperative tissue quality afer vaginal reconstructive surgery. Female Pelvic Med. Reconstr. Surg..

[24] Raghunandan C., Agrawal S., Dubey P., Choudhury M., Jain A. (2010). A comparative study of the effects of local estrogen with or without local testosterone on vulvovaginal and sexual dysfunction in postmenopausal women. J. Sex. Med..

[25] Daneshmand F., Hosseinzadeh P., Ghahiri A., Ghasemi M. (2014). A comparative study of vaginal estrogen cream and sustained released estradiol vaginal tablet (Vagifem) in the treatment of atrophic vaginitis among postmenopausal women. Iran. J. Reprod. Med..

[26] Dugal R., Hesla K., Sordal T., Aase K.H., Lilleeidet O., Wickstrom E. (2000). Comparisons of usefulness of estradiol vaginal tablets and estriol vagitories for treatment of vaginal atrophy. Acta Obstet. Et Gynecol. Scand..

[27] Barentsen R., Van de Weijer P.H.M., Schram J.H.N. (1997). Continuous low dose estradiol released from a vaginal ring versus estriol vaginal cream for urogenital atrophy. Eur. J. Obstet. Gynecol. Reprod. Biol..

[28] Casper F., Petri E. (1999). Local treatment of urogenital atrophy with an estradiol-releasing vaginal ring: A comparative and placebo controlled multicenter study. Int. Urogynecol. J..

[29] Iosif C.S., Batra S., Ek A., Astedt B. (1981). Estrogen receptors in the human female lower urinary tract. Am. J. Obstet. Gynecol..

[30] Al-Baghdadi O., Ewies A.A. (2009). Topical estrogen therapy in the management of postmenopausal vaginal atrophy: An up-to-date overview. Climacteric.

[31] Xie Z., Shi H., Zhou C., Xie Z., Shi H., Zhou C., Dong M., Hong L., Jin H. (2007). Alterations of estrogen receptor-alpha and -beta in the anterior vaginal wall of women with urinary incontinence. Eur. J. Obstet. Gynecol. Reprod. Biol..

[32] Cardozo L., Bachmann G., McClish D., Fonda D., Birgerson L. (1998). Meta-analysis of estrogen therapy in the management of urogenital atrophy in postmenopausal women: Second report of the Hormones and Urogenital Therapy Committee. Obstet. Gynecol..

[33] Jarmy-Di Bella Z.I., Girao M.J., Sartori M.F., Di Bella Junior V., Lederman H.M., Baracat E.C., Lima G.R. (2000). Power Doppler of the urethra in continent or incontinent, pre- and postmenopausal women. Int. Urogynecol. J. Pelvic Floor. Dysfunct.

[34] Rud T., Andersson K.E., Asmussen M., Hunting A., Ulmsten U. (1980). Factors maintaining the intraurethral pressure in women. Investig. Urol..

[35] Mulholland S.G., Qureshi S.M., Fritz R.W., Silverman H. (1982). Effect of hormonal deprivation on the bladder defense mechanism. J. Urol..

[36] Ballagh S.A. (2005). Vaginal hormone therapy for urogenital and menopausal symptoms. Semin. Reprod. Med..

[37] Rioux J.E., Devlin C., Gelfand M.M., Steinberg W.M., Hepburn D.S. (2000). 17beta-estradiol vaginal tablet versus conjugated equine estrogen vaginal cream to relieve menopausal atrophic vaginitis. Menopause.

[38] Ewies A.A.A., Alfhaily F. (2010). Topical vaginal estrogen therapy in managing postmenopausal urinary symptoms: A reality or a gimmick?. Climacteric.

[39] Davis S.R., McCloud P., Strauss B.J., Burger H. (1995). Testosterone enhances estradiol’s effects on postmenopausal bone density and sexuality. Maturitas.

[40] Simpson E.R. (2002). Aromatization of androgens in women: Current concepts and findings. Fertil. Steril..

[41] Webb S.J., Geoghegan T.E., Prough R.A., Miller K.K.M. (2006). The biological actions of dehydroepiandrosterone involves multiple receptors. Drug Metab. Rev..

[42] Maninger N., Wolkowitz O.M., Reus V.I., Epel E.S., Mellon S.H. (2009). Neurobiological and neuropsychiatric effects of dehydroepiandrosterone (DHEA) and DHEA sulfate (DHEAS). Front. Neuroendocrinol..

[43] Davison S.L., Davis S.R. (2003). Androgens in women. J. Steroid Biochem. Mol. Biol..

[44] Labrie F., Archer D., Bouchard C., Fortier M., Cusan L., Gomez J.-L., Girard G., Baron M., Ayotte N., Moreau M. (2010). High internal consistency and efficacy of intravaginal DHEA for vaginal atrophy. Gynecol. Endocrinol..

[45] Panjari M., Davis S.R. (2011). Vaginal DHEA to treat menopause related atrophy: A review of the evidence. Maturita.

[46] Labrie F., Archer D.F., Bouchard C., Girard G., Ayotte N., Gallagher J.C., Cusan L., Baron M., Blouin F., Waldbaum A.S. (2015). Prasterone has parallel beneficial effects on the main symptoms of vulvovaginal atrophy: 52-week open-label study. Maturitas.

[47] Berger L., El-Alfy M., Martel C., Labrie F. (2005). Effects of dehydroepiandrosterone, premarin and acolbifene on histomorphology and sex steroid receptors in the rat vagina. J. Steroid Biochem. Mol. Biol..

[48] Pelletier G., Ouellet J., Martel C., Labrie F. (2013). Androgenic action of dehydroepiandrosterone (DHEA) on nerve density in the ovariectomized rat vagina. J. Sex. Med..

[49] Labrie F., Archer D.F., Koltun W., Vachon A., Young D., Frenette L., Portman D., Montesino M., Côté I., Parent J. (2018). Efficacy of intravaginal dehydroepiandrosterone (DHEA) on moderate to severe dyspareunia and vaginal dryness, symptoms of vulvovaginal atrophy, and of the genitourinary syndrome of menopause. Menopause.

[50] Shufelt C.L., Braunstein G.D. (2009). Safety of testosterone use in women. Maturitas.

[51] Zang H., Sahlin L., Masironi B., Eriksson E., Linden Hirschberg A. (2007). Effects of testosterone treatment on endometrial proliferation in postmenopausal women. J. Clin. Endocrinol. Metab..

[52] Hodgson T.K., Braunstein G.D., Azziz R., Nestler J.E., Dewailly D. (2006). Physiological effects of androgens in women. Androgen Excess Disorders in Women: Polycystic Ovary Syndrome and Other Disorders.

[53] Wierman M.E., Arlt W., Basson R., Davis S.R., Miller K.K., Murad M.H., Rosner W., Santoro N. (2014). Androgen therapy in women: A reappraisal: An Endocrine Society clinical practice guideline. J. Clin. Endocrinol. Metab..

[54] European Medicines Agency (2019). Intrarosa: Summary of Product Characteristics. http://www.ema.europa.eu/.

[55] Labrie F., Archer D., Bouchard C., Fortier M., Cusan L., Gomez J.-L., Girard G., Baron M., Ayotte N., Moreau M. (2009). Effect of intravaginal dehydroepiandrosterone (Prasterone) on libido and sexual dysfunction in postmenopausal women. Menopause.

[56] Chee W.J.Y., Chew S.Y., Than L.T.L. (2020). Vaginal microbiota and the potential of Lactobacillus derivatives in maintaining vaginal health. Microb. Cell Factories.

[57] Smith S.B., Ravel J. (2017). The vaginal microbiota, host defence and reproductive physiology. J. Physiol..

[58] Gajer P., Brotman R.M., Bai G., Sakamoto J., Schütte U.M.E., Zhong X., Koenig S.S.K., Fu L., Ma Z., Zhou X. (2012). Temporal dynamics of the human vaginal microbiota. Sci. Transl. Med..

[59] Gupta S., Kakkar V., Bhushan I. (2019). Crosstalk between vaginal microbiome and female health: A review. Microb. Pathog..

[60] Torcia M.G. (2019). Interplay among vaginal microbiome, immune response and sexually transmitted viral infections. Int. J. Mol. Sci..

[61] Aldunate M., Srbinovski D., Hearps A.C., Latham C.F., Ramsland P.A., Gugasyan R., Cone R.A., Tachedjian G. (2015). Antimicrobial and immune modulatory effects of lactic acid and short chain fatty acids produced by vaginal microbiota associated with eubiosis and bacterial vaginosis. Front. Physiol..

[62] Barrientos-Durán A., Fuentes-López A., de Salazar A., Plaza-Díaz J., García F. (2020). Reviewing the Composition of Vaginal Microbiota: Inclusion of Nutrition and Probiotic Factors in the Maintenance of Eubiosis. Nutrients.

[63] Han Y., Liu Z., Chen T. (2021). Role of Vaginal Microbiota Dysbiosis in Gynecological Diseases and the Potential Interventions. Front. Microbiol..

[64] Lewis F.M., Bernstein K.T., Aral S.O. (2017). Vaginal microbiome and its relationship to behavior, sexual health, and sexually transmitted diseases. Obstet. Gynecol..

[65] Naumova I., Castelo-Branco C. (2018). Current treatment options for postmenopausal vaginal atrophy. Int. J. Women’s Health.

[66] Muhleisen A.L., Herbst-Kralovetz M.M. (2016). Menopause and the vaginal microbiome. Maturitas.

[67] Leyva-Gómez G., Del Prado-Audelo M.L., Ortega-Peña S., Mendoza-Muñoz N., Urbán-Morlán Z., González-Torres M., González-Del Carmen M., Figueroa-González G., Reyes-Hernández O.D., Cortés H. (2019). Modifications in Vaginal Microbiota and Their Influence on Drug Release. Chall. Oppor. Pharm..

[68] Mitchell C.M., Srinivasan S., Zhan X., Wu M.C., Reed S.D., Guthrie K.A., LaCroix A.Z., Fiedler T., Munch M., Liu C. (2017). Vaginal microbiota and genitourinary menopausal symptoms: A cross sectional analysis. Menopause.

[69] Amabebe E., Anumba D.O.C. (2018). The Vaginal Microenvironment: The Physiologic Role of Lactobacilli. Front. Med..

[70] Domoney C. (2014). Treatment of vaginal atrophy. Women’s Health.

[71] Marschalek M.-L., Bodner K., Kimberger O., Zehetmayer S., Morgenbesser R., Dietrich W., Obruca C., Husslein H., Umek H., Koelbl H. (2021). Does preoperative locally applied estrogen treatment facilitate prolapse-associated symptoms in postmenopausal women with symptomatic pelvic organ prolapse? A randomised controlled double-masked, placebo-controlled, multicenter study. Int. J. Obstet. Ang Gynecol..

[72] Zhixing S., Zhu L., Xu T., Shi X., Lang J. (2016). Effects of preoperative vaginal estrogen therapy for the incidence of mesh complication after pelvic organ prolapse surgery in postmenopausal women: Is it helpful or a myth? A 1-year randomized controlled trial. Menopause.

[73] Yu X., He L., Wang Y., Wang L., Yang Z., Lin Y. (2022). Local Estrogen Therapy for Pelvic Organ Prolapse in Postmenopausal Women: A Systematic Review and Meta-Analysis. Iran. J. Public Health.

[74] Loze Onwude J. (2009). Stress incontinence. BMJ Clin. Evid..

[75] Collà Ruvolo C., Gabrielli O., Formisano C., Califano G., Manna P., Venturella R., Di Carlo C. (2022). Prasterone in the treatment of mild to moderate urge incontinence: An observational study. Menopause.

[76] Hummelen R., Macklaim J.M., Bisanz J.E., Hammond J.A., McMillan A., Vongsa R., Koenig D., Gloor G.B., Reid G. (2011). Vaginal microbiome and epithelial gene array in postmenopausal women with moderate to severe dryness. PLoS ONE.

